# Developmental Versus Chromosomal Competence in Endometriosis: A Stepwise IVF Outcome Analysis

**DOI:** 10.3390/medicina62051001

**Published:** 2026-05-21

**Authors:** Luana Ghilea (Seleș), Viorela Romina Murvai, Patronela Naghi, Laura Maghiar, Alin Bodog, Carmen Anca Huniadi, Romeo Micu

**Affiliations:** 1 Department of Surgical Sciences, Obstetrics and Gynecology, Faculty of Medicine and Pharmacy, University of Oradea, 410087 Oradea, Romania; laura.maghiar@uoradea.ro (L.M.); alin.bodog@didactic.uoradea.ro (A.B.); ahuniadi@uoradea.ro (C.A.H.); 2 Doctoral School of Biological and Biomedical Sciences, University of Oradea, 1 University Street, 410087 Oradea, Romania; 3 Department of Obstetrics and Gynecology, Calla—Infertility Diagnostic and Treatment Center, Constantin A. Rosetti Street, 410103 Oradea, Romania; dr.rominamurvai@gmail.com; 4 Department of Embryology, Calla—Infertility Diagnostic and Treatment Center, Constantin A. Rosetti Street, 410103 Oradea, Romania; petronelanaghi@gmail.com; 5 Department of Obstetrics and Gynecology, University of Medicine and Pharmacy “Iuliu Hațieganu”, 400347 Cluj-Napoca, Romania; romeo.micu@umfcluj.ro

**Keywords:** endometriosis, IVF, embryo competence, blastocyst formation, aneuploidy, PGT-A, oocyte maturation, reproductive outcomes

## Abstract

*Background and Objectives*: Endometriosis is a multifactorial gynecological condition associated with impaired fertility; however, its impact on embryo competence remains incompletely understood. This study aimed to evaluate embryo competence through a stepwise analysis of IVF outcomes across the developmental continuum, while also comparing patients with endometriosis and controls. *Materials and Methods*: A retrospective observational study was conducted, including 160 patients undergoing IVF, comprising 55 patients with endometriosis and 105 controls. Clinical and embryological data were analyzed sequentially across key developmental stages, including oocyte retrieval, metaphase II (MII) oocyte formation, fertilization (2PN), embryo development, and euploidy in a subgroup undergoing preimplantation genetic testing for aneuploidy (PGT-A). Stage-specific efficiency rates were calculated, and correlations between early- and late-developmental parameters were assessed. In addition, comparative analysis between groups was performed. *Results*: A progressive decline in developmental efficiency was observed across the IVF continuum, with approximately one-quarter of retrieved oocytes reaching the embryo stage and only a small proportion ultimately resulting in euploid Blastocysts. Strong positive correlations were identified among early-stage parameters, particularly retrieved oocytes, MII oocytes, and embryo yield (r = 0.77–0.96, *p* < 0.001), indicating that ovarian response and oocyte maturity significantly influence downstream outcomes. However, efficiency-based parameters showed limited predictive value for chromosomal competence. A moderate association was observed between MII oocytes and euploid Blastocysts (r = 0.58), whereas the relationship between embryo number and euploidy remained weak. Comparative analysis revealed no statistically significant differences between the endometriosis and control groups across the evaluated embryological parameters (*p* > 0.05 for all comparisons), suggesting that sequential analyses may provide complementary insight beyond direct comparisons. *Conclusions*: IVF outcomes follow a sequential developmental trajectory with a progressive decline in efficiency across stages. In endometriosis, early developmental competence appears to be affected, while chromosomal competence remains relatively preserved.

## 1. Introduction

Endometriosis is a multifaceted gynecological condition driven by chronic inflammation, hormonal dysregulation, and ectopic endometrial tissue implantation, with significant implications for female reproductive function [1,2]. Despite extensive research, the mechanisms by which endometriosis impairs reproductive outcomes remain incompletely understood. The disease is characterized by ectopic endometrial tissue, chronic inflammation, oxidative stress, and altered hormonal signaling, all of which may negatively influence oocyte quality, embryo development, and endometrial receptivity [2,3].

In assisted reproductive technologies (ART), particularly in vitro fertilization (IVF), reproductive success depends on a sequence of tightly regulated biological events that can be conceptualized as a developmental continuum: oocyte retrieval → oocyte maturation (MII) → fertilization → Blastocyst formation → chromosomal competence (euploidy). Each step represents a critical checkpoint, and impairment at any level may reduce the likelihood of achieving a viable pregnancy. Evaluating these stages individually may provide only partial information, whereas analyzing them sequentially allows for a more comprehensive understanding of embryo competence.

The impact of endometriosis on IVF outcomes has been widely investigated; however, findings remain inconsistent. Several studies have reported reduced oocyte yield, impaired fertilization rates, and decreased Blastocyst formation, suggesting compromised developmental competence [3,4]. Conversely, other reports have shown comparable embryo development outcomes after adjustment for confounding variables such as maternal age and ovarian reserve [5]. These discrepancies indicate that the effect of endometriosis may not be uniform across all stages of embryo development and highlight the need for a stepwise analytical approach.

A key area of ongoing debate is whether endometriosis affects embryo competence at the level of chromosomal integrity or primarily interferes with early developmental processes. The introduction of preimplantation genetic testing for aneuploidy (PGT-A) has enabled more precise assessment of the chromosomal status of Blastocysts; however, current evidence suggests that aneuploidy rates among patients with endometriosis may be comparable to those in control groups [6,7,8]. This raises the possibility that the detrimental effects of endometriosis are more pronounced during earlier stages of the developmental trajectory rather than at the level of chromosomal competence.

Moreover, alterations in the follicular microenvironment, including increased inflammatory mediators and oxidative stress, have been proposed as mechanisms through which endometriosis may impair oocyte competence and early embryogenesis [3]. Such changes may disrupt the progression along the developmental axis without necessarily inducing chromosomal abnormalities, thereby affecting the efficiency of embryo development rather than its genetic integrity.

Therefore, evaluating reproductive outcomes across the entire developmental pathway from oocyte maturation to euploid Blastocyst formation may provide a more accurate, mechanistic understanding of endometriosis’s impact. A stepwise analysis of this axis allows differentiation between developmental competence (the ability to progress through successive stages) and chromosomal competence (the ability to maintain genetic integrity).

Although previous studies have investigated developmental kinetics, embryo competence, and aneuploidy in IVF, integrated stepwise analyses evaluating sequential developmental progression from oocyte maturation to euploidy remain relatively limited.

This study aimed to assess embryonic competence through a stepwise analysis of IVF outcomes, from oocyte maturation to euploidy, and to compare patients with endometriosis to controls. We hypothesized that endometriosis predominantly affects early developmental efficiency while having limited impact on the chromosomal status of resulting Blastocysts. These findings may help clarify the pathophysiology of endometriosis-related infertility and inform clinical decision-making in assisted reproduction.

## 2. Materials and Methods

### 2.1. Study Design and Population

This retrospective observational study included 160 patients who underwent in vitro fertilization (IVF) cycles at the Calla IVF Center between January 2023 and January 2026. Only one IVF cycle per patient was included in the analysis to preserve statistical independence. Ethical approval for the retrospective analysis of previously collected clinical and embryological data was obtained from the Institutional Review Board of the Calla IVF Center (No. 3678/A, 5 January 2026). The study was conducted in accordance with the Declaration of Helsinki.

The study population comprised patients with complete embryological records, including retrieved oocytes, MII oocytes, fertilization outcomes (2PN), embryo development, and, where applicable, PGT-A results, allowing a comprehensive evaluation of reproductive outcomes across all IVF stages. For comparative purposes, patients were divided into two groups: those diagnosed with endometriosis (*n* = 55) and a control group without endometriosis (*n* = 105).

In addition, a subgroup of 18 patients undergoing preimplantation genetic testing for aneuploidy (PGT-A) was evaluated separately to assess chromosomal competence. Within this subgroup, a total of 41 Blastocysts were biopsied, of which 12 were classified as euploid.

The diagnosis of endometriosis was established based on clinical evaluation, transvaginal ultrasonography, and/or previous laparoscopic or surgical confirmation, according to standard gynecological practice.

Controlled ovarian stimulation protocols were individualized according to patient age, ovarian reserve, and clinical characteristics, using standard gonadotropin-based antagonist protocols routinely applied in the IVF center.

Inclusion criteria were defined as the availability of complete clinical and embryological records, allowing assessment of oocyte retrieval, oocyte maturation, fertilization, embryo development, and, where applicable, chromosomal competence. Cycles with incomplete data or missing key embryological parameters were excluded from the analysis.

### 2.2. Data Collection and Variables

Clinical and embryological data were collected from electronic medical records and laboratory databases of Calla IVF Center. The variables analyzed included the number of retrieved oocytes, the number of mature oocytes (MII), the number of fertilized oocytes (2PN), and the number of Blastocysts obtained.

In addition, a subgroup of patients undergoing preimplantation genetic testing for aneuploidy (PGT-A) was evaluated separately to assess chromosomal competence, using the numbers of biopsied and euploid Blastocysts. Maternal age was also recorded as a key clinical variable due to its established impact on reproductive outcomes.

### 2.3. Definition of the Developmental Continuum and Efficiency Rates

The IVF process was conceptualized as a sequence of successive stages: oocyte retrieval, oocyte maturation (MII), fertilization (2PN), embryo development, and euploidy. To evaluate biological efficiency across these stages, specific rates were calculated. The MII rate was defined as the ratio of mature oocytes to retrieved oocytes, the fertilization rate as the ratio of fertilized oocytes (2PN) to MII oocytes, and the Blastulation rate as the number of Blastocysts relative to the number of fertilized oocytes. Finally, the euploidy rate was calculated as the proportion of euploid Blastocysts among biopsied Blastocysts.

This stepwise framework enabled the evaluation of both developmental competence, which reflects the ability to progress through successive stages, and chromosomal competence, which reflects the genetic integrity of Blastocysts.

### 2.4. Statistical Analysis

Statistical analysis was performed using IBM SPSS Statistics version 26.0 (IBM Corp., Armonk, NY, USA). Continuous variables were expressed as mean ± standard deviation (SD). Normality of data distribution was assessed using the Shapiro–Wilk test. For the comparative analysis between the endometriosis and control groups, variables demonstrating normal distribution were analyzed using the independent samples t-test, whereas variables violating normality assumptions were analyzed using the Mann–Whitney U test. Pearson’s correlation coefficient (r) was used to assess linear relationships between variables across different IVF stages. Correlation strength was categorized as weak (r < 0.3), moderate (0.3–0.6), or strong (>0.6). A *p*-value < 0.05 was considered statistically significant.

## 3. Results

**Figure 1:**
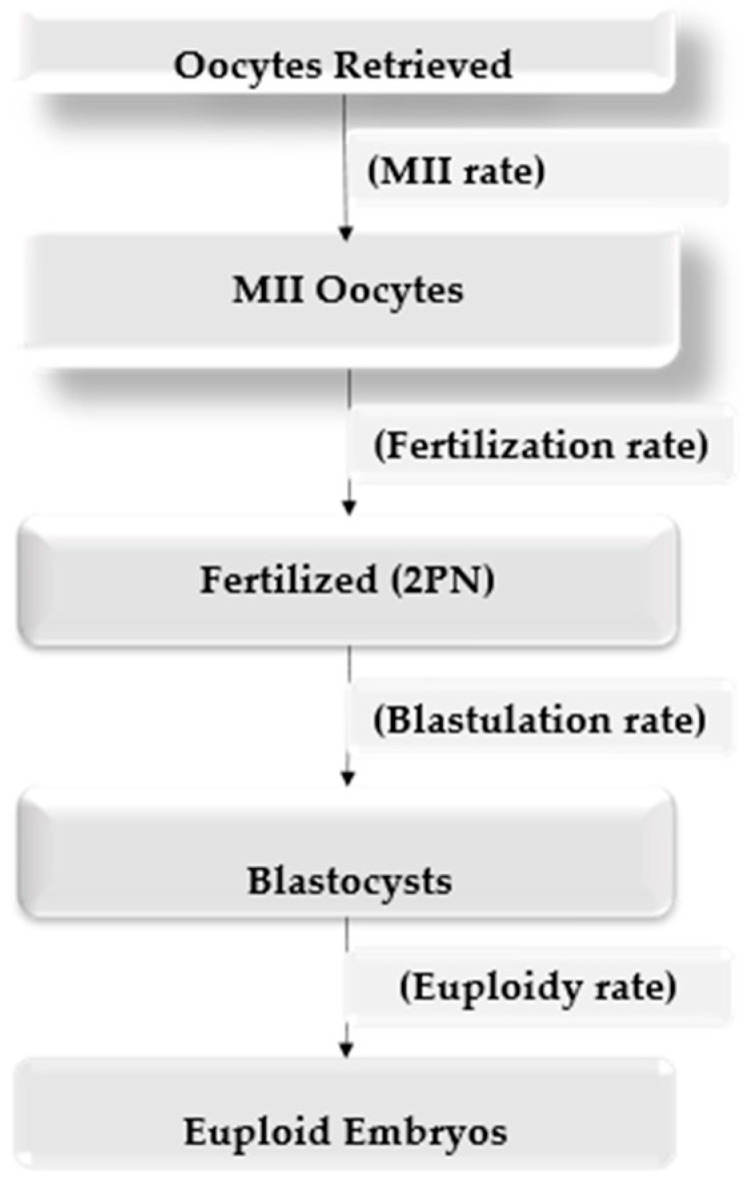
Study workflow illustrating sequential IVF stages and stage-specific efficiency rates.

The process follows a sequence of stages from oocyte retrieval to euploid embryo formation, with stage-specific efficiency rates indicated between consecutive steps (Figure 1).

### 3.1. Baseline Characteristics of the Study Population

The study population included women undergoing in vitro fertilization (IVF) cycles, with baseline demographic and clinical characteristics summarized in Table 1 to provide context for subsequent analyses.

The mean age of the patients was 36.06 ± 4.20 years, reflecting a cohort predominantly within the late reproductive age range, which is particularly relevant given the known influence of maternal age on oocyte quality and chromosomal competence. No statistically significant differences in age distribution were observed between patients with endometriosis and controls.

Ovarian response, assessed by the number of retrieved oocytes, showed considerable variability, with a mean of 8.95 ± 7.91 oocytes. The number of mature oocytes (MII) was 6.11 ± 5.03, indicating variability in oocyte maturation across cycles. These parameters were comparable between groups, with no statistically significant differences identified.

Fertilization outcomes resulted in a mean of 4.74 ± 3.90 fertilized oocytes, while embryo development yielded an average of 2.27 ± 2.11 Blastocysts per cycle. Similarly, no significant differences were observed between the endometriosis and control groups for these parameters.

Overall, the baseline characteristics demonstrate substantial inter-individual variability in ovarian response and embryological outcomes, without significant differences between groups. The progressive decrease in cell number from oocyte retrieval to embryo formation reflects the cumulative biological attrition inherent to the IVF process.

Detailed baseline characteristics are presented in Table 1.

### 3.2. Sequential Embryological Outcomes and Developmental Efficiency

#### 3.2.1. Relationship Between Ovarian Response and Oocyte Maturation

The relationship between ovarian response and oocyte maturation was first evaluated. A strong positive correlation was observed between the number of retrieved oocytes and the number of MII oocytes (r = 0.96, *p* < 0.001; Table 2), indicating that increased ovarian response is directly associated with a higher yield of mature oocytes.

This finding supports the concept that the efficiency of ovarian stimulation plays a critical role in determining the initial pool of developmentally competent oocytes. However, the observed inter-individual variability suggests that not all retrieved oocytes achieve full maturation, highlighting the influence of intrinsic oocyte quality.

The strong positive correlation observed in our study is consistent with previous reports; however, the magnitude of the association (r = 0.96) highlights the robustness of the relationship within our cohort.

#### 3.2.2. Association Between Oocyte Maturity and Fertilization Outcomes

The impact of oocyte maturity on fertilization success was further analyzed. A strong positive correlation was identified between the number of MII oocytes and the number of fertilized oocytes (2PN) (r = 0.97, *p* < 0.001; Table 3), demonstrating that oocyte maturity is a key determinant of successful fertilization.

These results indicate that the transition from immature to mature oocytes represents a critical biological checkpoint within the IVF continuum, directly influencing downstream developmental competence.

The strength of this association highlights oocyte maturity as a central determinant of fertilization efficiency within the IVF process.

#### 3.2.3. Fertilization and Embryo Development

Embryo development was evaluated in relation to fertilization outcomes. A strong positive correlation was observed between the number of fertilized oocytes and the number of Blastocysts obtained (r = 0.77, *p* < 0.001; Table 4), indicating that fertilization success significantly influences downstream embryological development.

Although the strength of the association remained high, it was lower than in the previous stages, reflecting the increasing biological complexity and attrition during early embryogenesis.

The progressive reduction in cell numbers from fertilization to embryo formation is consistent with the expected biological attrition inherent to early developmental processes.

The gradual decrease in correlation strength across stages reflects the increasing biological variability and selection processes occurring during early embryonic development.

#### 3.2.4. Analysis of Stage-Specific Efficiency Rates

To further characterize the IVF process, stage-specific efficiency rates were analyzed across successive stages.

The mean MII rate was 0.76 ± 0.26, indicating that approximately three-quarters of retrieved oocytes reached full maturity. The fertilization rate was 0.73 ± 0.24, suggesting that the majority of mature oocytes successfully underwent fertilization. In contrast, the embryo development rate was lower, with a mean of 0.48 ± 0.33, reflecting increased biological attrition during early embryogenesis.

These findings demonstrate a progressive decline in efficiency across successive developmental stages, supporting the notion that biological selection intensifies as development advances. While early stages are predominantly influenced by quantitative factors, later stages appear to be increasingly governed by qualitative determinants, including oocyte competence and embryonic viability.

Overall, the analysis of stage-specific efficiency rates provides an integrated assessment of reproductive performance, demonstrating that success in IVF depends not only on the number of retrieved oocytes but also on the efficiency with which they progress through each developmental stage (Table 5).

When considered cumulatively, these stage-specific efficiencies yielded an overall developmental efficiency of approximately 0.27, indicating that only about one-quarter of retrieved oocytes ultimately progressed to the embryo stage. This finding highlights the substantial biological attrition that occurs across sequential embryological transitions.

Notably, the most pronounced decline in efficiency was observed during the transition from fertilization to embryo formation, with rates decreasing from 0.73 to 0.48, indicating that early embryogenesis represents a major biological bottleneck in IVF.

#### 3.2.5. Subanalysis of Chromosomal Competence (Euploidy)

Beyond morphological development, the final stage of the IVF continuum involves the acquisition of chromosomal competence.

A subgroup of patients (*n* = 18) underwent preimplantation genetic testing (PGT), enabling assessment of the embryonic genetic status (Table 6). Within this subgroup, variability was observed in both the number of Blastocysts available for biopsy and the proportion of euploid Blastocysts. The overall euploidy rate was 0.29, reflecting the expected heterogeneity within the studied cohort.

A moderate positive correlation was identified between the number of MII oocytes and the number of euploid Blastocysts (r = 0.58, *p* = 0.015), suggesting a potential relationship between oocyte maturity and genetic integrity. In contrast, the association between the total number of Blastocysts and euploid embryo yield was not statistically significant (r = 0.46, *p* = 0.08), indicating a weaker and more variable relationship at this stage.

These findings suggest that the IVF process may extend beyond morphological outcomes, with potential implications for the genetic quality of resulting Blastocysts. However, given the limited sample size, these observations should be interpreted with caution and considered exploratory.

When considering chromosomal competence, the cumulative probability of obtaining a euploid embryo from retrieved oocytes appears low, estimated at approximately 0.08, reflecting the combined impact of sequential biological attrition and genetic selection across IVF stages.

#### 3.2.6. Influence of Maternal Age on Developmental Outcomes

Maternal age was further analyzed in relation to key embryological outcomes across the IVF developmental continuum.

A statistically significant negative correlation was observed between maternal age and ovarian response, as reflected by the number of retrieved oocytes (r = −0.32, *p* = 0.026). Similarly, age showed a significant inverse association with the number of MII oocytes (r = −0.29, *p* = 0.045), suggesting a decline in oocyte maturation efficiency with increasing age.

In addition, maternal age was negatively correlated with the number of fertilized oocytes (r = −0.29, *p* = 0.040). A similar trend was observed for embryo yield (r = −0.27); however, this association did not reach statistical significance (*p* = 0.065).

These results are summarized in Table 7.

These findings support the role of maternal age as a relevant biological factor influencing ovarian response and early embryological outcomes. The observed decline across stages suggests that age-related effects may influence multiple IVF stages; however, the association between maternal age and embryo yield did not reach statistical significance.

In addition to absolute embryological counts, maternal age may also influence stage-specific efficiency rates, suggesting that reproductive aging affects not only the quantity of oocytes and Blastocysts, but also the probability of successful progression between consecutive developmental stages.

### 3.3. Integrated and Cross-Stage Analysis of Developmental Outcomes

#### 3.3.1. Cross-Stage Correlations Across the IVF Developmental Continuum

To further investigate the interdependence between early and late stages of the IVF process, cross-stage correlations were analyzed across the developmental continuum (Table 8).

A strong positive correlation was observed between the number of retrieved oocytes and the number of Blastocysts obtained (r = 0.68, *p* < 0.001), as well as between MII oocytes and embryo yield (r = 0.72, *p* < 0.001). Correlations involving euploid Blastocysts were calculated within the PGT-A subgroup (*n* = 18). Importantly, the number of retrieved oocytes was strongly associated with the number of euploid Blastocysts (r = 0.70, *p* < 0.001), indicating that ovarian response substantially impacts final genetic outcomes. MII oocytes showed a moderate positive correlation with euploid Blastocysts (r = 0.58, *p* = 0.015), while the relationship between total Blastocysts and euploid embryo yield remained weaker and did not reach statistical significance (r = 0.46, *p* = 0.08), suggesting increased variability and the influence of additional biological selection mechanisms at later stages.

Overall, these findings support the concept that early-stage parameters, particularly ovarian response and oocyte maturity, have a measurable impact on final embryological and genetic outcomes, although this influence diminishes as developmental complexity increases.

#### 3.3.2. Efficiency-Based Correlations

To further explore the role of biological efficiency across the IVF developmental continuum, correlations between stage-specific efficiency rates were analyzed (Table 9).

Overall, weak and non-significant associations were observed between efficiency parameters across consecutive stages. The MII rate showed only a minimal positive relationship with Blastulation rate (r = 0.10, *p* > 0.05), while the fertilization rate demonstrated a similarly weak association (r = 0.10, *p* > 0.05), indicating limited interdependence between efficiency at successive developmental steps.

Interestingly, MII rate exhibited a moderate negative correlation with euploidy rate (r = −0.38, *p* = 0.12), suggesting that higher maturation efficiency does not necessarily translate into improved chromosomal competence. Similarly, fertilization rate showed a weak negative relationship with euploidy rate (r = −0.12, *p* = 0.63).

Taken together, these findings indicate that efficiency-based parameters have limited predictive value for downstream developmental and genetic outcomes, highlighting the complex, multifactorial nature of embryonic competence.

These observations suggest that, while efficiency at individual stages may contribute to overall reproductive performance, it does not fully account for the variability observed in final embryological and genetic outcomes, reinforcing the role of intrinsic biological factors across the IVF continuum.

Taken together, these findings support the concept of IVF as a sequential developmental process in which early-stage quantitative parameters, particularly ovarian response and oocyte maturation, strongly influence downstream embryological outcomes, while chromosomal competence remains only partially explained, reflecting the complex, multifactorial nature of embryo quality.

### 3.4. Comparative Analysis Between Endometriosis and Control Groups

To complement the stepwise analysis, a direct comparison between patients with endometriosis and controls was conducted to assess potential differences in embryological outcomes along the IVF developmental continuum.

As shown in Table 10, no statistically significant differences were observed between the two groups for any of the analyzed embryological parameters. Age distribution was comparable, minimizing the potential confounding effect of maternal age on reproductive outcomes.

Data are presented as mean ± standard deviation (SD). Differences between groups were analyzed using an independent samples *t*-test or Mann–Whitney U test, as appropriate. A *p*-value < 0.05 was considered statistically significant. No statistically significant differences were observed across the analyzed parameters.

Similarly, ovarian response, reflected by the number of retrieved oocytes, did not differ significantly between groups. The number of MII oocytes and fertilized oocytes (2PN) also showed comparable values, suggesting that oocyte maturation and fertilization processes are not markedly impaired when assessed through direct group comparison.

Embryo yield showed similar distributions in the endometriosis and control groups, further supporting the absence of significant quantitative differences in early embryological outcomes.

Taken together, these findings indicate that conventional group comparisons may not fully reflect the complexity of sequential developmental dynamics across the IVF continuum. In contrast, the stepwise analysis presented in the previous sections reveals a progressive decline in developmental efficiency and highlights stage-specific vulnerabilities that are not evident when only absolute values are compared. This discrepancy underscores the importance of evaluating IVF outcomes across sequential stages, as stepwise analytical frameworks may provide additional insight into developmental dynamics beyond static group comparisons.

## 4. Discussion

The present study provides a stepwise evaluation of IVF outcomes along the developmental continuum, offering a mechanistic perspective on embryonic competence. In parallel, a direct comparison between patients with endometriosis and controls was performed to provide clinical context. While comparative analyses offer useful information, the stepwise framework enables the identification of stage-specific biological checkpoints that may be relevant for understanding developmental dynamics in conditions such as endometriosis.

A key finding of this study is the progressive decline in efficiency across sequential developmental stages, from oocyte retrieval to embryo formation and ultimately to chromosomal competence. In our cohort, only approximately one-quarter of retrieved oocytes progressed to the embryo stage, while only a small proportion ultimately resulted in euploid Blastocysts. These findings are consistent with previous studies demonstrating that embryo development is characterized by progressive biological attrition and selection mechanisms [9,10,11].

Across the overall study population, the most pronounced efficiency decline was observed during the transition from fertilization to embryo formation, indicating that early embryogenesis represents a critical biological bottleneck. This observation aligns with previous research showing that embryo developmental kinetics and morphology reflect underlying biological competence but do not fully capture developmental potential [12,13,14,15].

From a pathophysiological perspective, this stage may represent a biologically vulnerable phase in endometriosis. The disease is associated with chronic inflammation, oxidative stress, and alterations in the follicular microenvironment, which have been shown to impair oocyte competence and early embryonic development [16,17]. These mechanisms could potentially influence early developmental progression without necessarily affecting downstream chromosomal integrity.

The strong correlations identified between retrieved oocytes, MII oocytes, and embryo yield further emphasize the importance of early-stage quantitative parameters. These findings indicate that ovarian response and oocyte maturity play a central role in determining downstream developmental outcomes. Previous studies have reported that endometriosis is associated with altered oocyte yield and embryo development, although such differences were not statistically significant in our cohort [18,19,20].

Recent research investigating embryo competence and selection strategies has further emphasized the complexity of predicting reproductive outcomes, particularly when relying on static morphological or quantitative parameters [21,22,23,24]. In this context, embryonic development should be viewed as a dynamic, multifactorial process influenced by both intrinsic and extrinsic factors.

At the same time, emerging evidence highlights that oocyte quality and early developmental dynamics are key determinants of embryo competence, even in the absence of overt chromosomal abnormalities [25,26,27,28]. These findings may suggest that early developmental impairment could contribute to reproductive variability associated with endometriosis.

However, the relationship between developmental competence and embryo genetic status remains complex. In our study, efficiency-based parameters did not reliably predict euploidy outcomes, consistent with previous reports indicating that embryo morphology and morphokinetic parameters have limited predictive value for chromosomal status [22].

The low cumulative probability of obtaining a euploid embryo observed in our cohort reflects the combined effects of sequential biological attrition and genetic selection. This observation is consistent with previous studies showing that euploidy is a highly selective endpoint influenced by intrinsic embryonic factors, including oocyte quality and cellular integrity [29,30]. However, these results should be interpreted with caution, given the relatively small size of the PGT-A subgroup (*n* = 18), which may limit statistical power and reduce the generalizability of these findings.

Importantly, clinical evidence indicates that aneuploidy rates in patients with endometriosis are comparable to those observed in age-matched controls [29]. Due to the limited size of the PGT-A subgroup, subgroup-specific comparisons of euploidy rates between endometriosis and control patients were considered exploratory and were not sufficiently powered for definitive statistical interpretation. Therefore, conclusions regarding chromosomal competence should be interpreted cautiously. Our findings remain consistent with previous observations suggesting comparable aneuploidy rates between patients with endometriosis and controls.

In addition, the absence of statistically significant differences observed in direct comparisons between patients with endometriosis and controls further highlights the complexity of interpreting IVF outcomes in endometriosis. While absolute embryological parameters appeared comparable between groups, the stepwise analysis provided additional insight into the sequential attrition occurring across IVF stages. This finding highlights the limitations of static group comparisons in complex biological systems such as embryo development.

The analysis of efficiency-based correlations in our study further reinforces this interpretation. The weak and non-significant relationships observed between MII rate, fertilization rate, and downstream outcomes indicate that biological efficiency is not a linear process. In other words, favorable performance at one stage does not guarantee success at subsequent stages, supporting the concept that embryo development is governed by stage-specific regulatory mechanisms.

Maternal age was also identified as a relevant factor influencing multiple stages of IVF. The negative correlations observed across ovarian response, oocyte maturation, and fertilization outcomes are consistent with previous evidence demonstrating that reproductive aging affects both quantitative and qualitative aspects of oocyte competence [6]. Although inflammatory and oxidative mechanisms associated with endometriosis may contribute to reproductive impairment, the present study did not demonstrate statistically significant group-specific effects. In patients with endometriosis, these effects may be further amplified by inflammatory and oxidative processes affecting the ovarian environment [16,17].

Taken together, these findings highlight the sequential nature of developmental attrition across IVF stages and support the utility of stepwise analytical models for evaluating embryological outcomes. Although no statistically significant differences were identified between endometriosis and control groups, the proposed framework may help explore stage-specific developmental dynamics in future studies.

From a clinical perspective, these results suggest that strategies aimed at improving early developmental stages, particularly oocyte quality and embryo development, may be more effective than approaches focused exclusively on chromosomal selection. However, additional studies with larger cohorts and more extensive PGT-A analyses are necessary to clarify the specific impact of endometriosis on developmental and chromosomal competence. The stepwise analytical framework applied in this study provides a more comprehensive understanding of embryo competence and may contribute to optimizing clinical management in patients with endometriosis undergoing assisted reproductive treatments.

Several limitations of this study should be acknowledged. First, the stepwise analytical framework, although useful for capturing the sequential nature of IVF outcomes, simplifies a complex biological process and may not fully account for dynamic interactions between developmental stages. Second, potential confounding factors, including ovarian reserve markers (e.g., AMH), detailed endometriosis phenotypes and staging, prior surgical history, stimulation protocols, and laboratory conditions, were not fully controlled for, which may have influenced the observed associations. In addition, the unequal group sizes may have reduced the statistical power to detect subtle but potentially clinically relevant differences between endometriosis and control patients.

Finally, the relatively small size of the PGT-A subgroup limits the generalizability of conclusions regarding chromosomal competence.

## 5. Conclusions

IVF outcomes should be interpreted as a sequential developmental continuum characterized by progressive biological attrition. While no statistically significant differences were observed between patients with endometriosis and controls, the stepwise analytical framework may offer a complementary perspective on the sequential developmental dynamics occurring throughout the IVF process. However, the present data do not definitively demonstrate a differential effect of endometriosis on developmental versus chromosomal competence.

## Figures and Tables

**Table 1:** Baseline characteristics.

Variable	Total (*n* = 160)	Endometriosis (*n* = 55)	Control (*n* = 105)	*p*-Value
Age (years)	36.06 ± 4.20	36.2 ± 4.1	35.9 ± 4.3	0.68
Retrieved oocytes (*n*)	8.95 ± 7.91	9.3 ± 8.1	8.7 ± 7.8	0.74
MII oocytes (*n*)	6.11 ± 5.03	6.4 ± 5.2	5.9 ± 4.9	0.65
Fertilized oocytes (2PN) (*n*)	4.74 ± 3.90	4.9 ± 4.1	4.6 ± 3.8	0.72
Blastocysts (*n*)	2.27 ± 2.11	2.3 ± 2.2	2.2 ± 2.0	0.81

**Table 2:** Correlation between ovarian response and oocyte maturation.

Variables	r (Pearson)	*p*-Value
Retrieved oocytes vs. MII oocytes	0.96	<0.001

**Table 3:** Correlation between oocyte maturity and fertilization outcomes.

Variables	r (Pearson)	*p*-Value
MII oocytes vs. fertilized oocytes (2PN)	0.97	<0.001

**Table 4:** Correlation between fertilization outcomes and embryo development.

Variables	r (Pearson)	*p*-Value
Fertilized oocytes (2PN) vs. Blastocysts	0.77	<0.001

**Table 5:** Stage-specific efficiency rates across IVF stages.

Rate	Mean ± SD
MII rate	0.76 ± 0.26
Fertilization rate	0.73 ± 0.24
Blastulation rate	0.48 ± 0.33

**Table 6:** Genetic outcomes in the PGT subgroup.

Parameter	Value
Patients (*n*)	18
Biopsied Blastocysts (*n*)	41
Euploid Blastocysts (*n*)	12
Euploidy rate	0.29

**Table 7:** Correlation between maternal age and embryological outcomes.

Variables	r (Pearson)	*p*-Value
Age vs. retrieved oocytes	−0.32	0.026
Age vs. MII oocytes	−0.29	0.045
Age vs. fertilized oocytes (2PN)	−0.29	0.040
Age vs. Blastocysts	−0.27	0.065

**Table 8:** Cross-stage correlations across IVF stages.

Variables	r (Pearson)	*p*-Value
Retrieved oocytes vs. Blastocysts	0.68	<0.001
MII oocytes vs. Blastocysts	0.72	<0.001
Retrieved oocytes vs. euploid Blastocysts	0.70	<0.001
MII oocytes vs. euploid Blastocysts	0.58	0.015
Blastocysts vs. euploid Blastocysts	0.46	0.08

**Table 9:** Correlation between stage-specific efficiency rates.

Variables	r (Pearson)	*p*-Value
MII rate vs. Blastulation rate	0.10	>0.05
MII rate vs. euploidy rate	−0.38	0.12
Fertilization rate vs. Blastulation rate	0.10	>0.05
Fertilization rate vs. euploidy rate	−0.12	0.63

**Table 10:** Comparison of embryological outcomes between endometriosis and control groups.

Variable	Endometriosis (*n* = 55)	Control (*n* = 105)	*p*-Value
Age (years)	36.2 ± 4.1	35.9 ± 4.3	0.68
Retrieved oocytes (*n*)	9.3 ± 8.1	8.7 ± 7.8	0.74
MII oocytes (*n*)	6.4 ± 5.2	5.9 ± 4.9	0.65
Fertilized oocytes (2PN) (*n*)	4.9 ± 4.1	4.6 ± 3.8	0.72
Blastocysts (*n*)	2.3 ± 2.2	2.2 ± 2.0	0.81

## Data Availability

The data presented in this study are available on reasonable request from the corresponding author. The data are not publicly available due to privacy and ethical restrictions.
